# Angiotensin-converting enzyme inhibitors for aortic stenosis: a drug-target Mendelian randomization study

**DOI:** 10.1016/j.ijcha.2025.101729

**Published:** 2025-06-19

**Authors:** Jonathan L. Ciofani, Daniel Han, Benjamin Woolf, Dipender Gill, Usaid K. Allahwala, Ravinay Bhindi

**Affiliations:** Sydney Medical School, The University of Sydney, Sydney, Australia; Department of Cardiology, Royal North Shore Hospital, Sydney, Australia; Department of Epidemiology and Biostatistics, School of Public Health, Imperial College London, London, UK; School of Mathematics and Statistics, University of New South Wales, Sydney, Australia; School of Psychological Science, University of Bristol, Bristol, UK; MRC Integrative Epidemiology Unit, University of Bristol, Bristol, UK; MRC Biostatistics Unit, University of Cambridge, Cambridge, UK; Department of Epidemiology and Biostatistics, School of Public Health, Imperial College London, London, UK; Sydney Medical School, The University of Sydney, Sydney, Australia; Department of Cardiology, Royal North Shore Hospital, Sydney, Australia; Sydney Medical School, The University of Sydney, Sydney, Australia; Department of Cardiology, Royal North Shore Hospital, Sydney, Australia

**Correspondence**

Calcific aortic stenosis (AS) continues to rise in prevalence yet there remains no evidence-based medical therapy. The American guidelines for valvular heart disease highlight hypertension as a potential AS risk factor, and specifically identifies renin-angiotensin system blockade as a potential therapeutic strategy to improve outcomes after transcatheter aortic valve implantation [1]. However, there is no robust evidence to guide antihypertensive therapy for the prevention of AS development or progression. Drug-target Mendelian randomization (MR) can predict drug effects before committing to costly and time-consuming randomized controlled trials [2]. This study applies drug-target MR to evaluate the role of angiotensin converting enzyme (ACE) inhibition on risk of AS.

Conventional MR analyses were performed to evaluate for a potentially causal relationship between systolic blood pressure (SBP) and AS risk, using previously described methods [3]. Then drug-target MR was performed using distal and proximal effects approaches. The distal effects approach employs genetic variants in or near the drug-target gene that influence downstream biomarkers – in this case, using *ACE* gene variants that affect SBP to capture the physiological consequences of ACE inhibition. The proximal effects approach utilises genetic variants that directly modulate the expression of the drug target gene itself. We prioritised the distal SBP approach as our primary analysis given its superior statistical power with greater sample size and established clinical relevance, while the proximal approach provides corroborating genetic evidence. For both the conventional and drug-target MR approaches, data for SBP was obtained from a GWAS of up to 1,028,980 participants of European ancestry [4], for AS from a GWAS cohort including 14,819 cases and 941,863 controls [5], and a control GWAS for coronary artery disease from a cohort of 60,801 cases and 123,504 controls [6]. The distal effect approach identified single nucleotide polymorphisms in or within 200 kb of the *ACE* gene associated with SBP at p < 5 × 10^−8^. Palindromic SNPs were excluded and clumping thresholds of 1 Mb distance and r^2^ < 0.01 for linkage disequilibrium were applied using the 1000 Genomes Phase 3 European reference panel. The primary analysis was Wald ratio, and colocalisation analysis performed. For validation, we proxied ACE inhibition by its proximal effects. Data from the eQTLGen Consortium [7] was used to identify variants associated with *ACE* gene expression for evaluation in summary-data based Mendelian randomization (SMR) and heterogeneity in dependent instruments (HEIDI) analyses. Statistical analyses were performed using R version 4.4.2 and the SMR software package.

Genetically predicted higher SBP was associated with increased risk of AS (OR 1.03 per mmHg increase in SBP, 95 %CI 1.02–1.04, p < 0.0001). This was consistent on all MR sensitivity analyses (Fig. 1A). One SNP (rs2286526) was identified in the *ACE* gene region that was significantly associated with SBP. To evaluate whether the association between SBP and AS was solely driven by the ACE-region variant we performed leave-one-out analysis excluding rs2286526, which showed virtually identical results (OR 1.03, 95 % CI 1.02–1.04, p < 0.0001). On Wald ratio analysis, genetically predicted ACE inhibition was significantly associated with lower AS risk (OR 0.92 per mmHg lower SBP, 95 %CI 0.85–0.99, p = 0.027) (Fig. 1B). Enumeration colocalisation was underpowered for further evaluation (sum of the posterior probabilities of H3 and H4 < 0.1). Validation analysis evaluating the proximal effects of *ACE* perturbation demonstrated concordant results. SMR analysis using eQTL data showed significantly lower AS risk (OR 0.69 per SD decrease in gene expression, 95 %CI 0.49–0.98, p = 0.041), with no evidence of multiple causal variants (HEIDI analysis p = 0.57). Validation analysis also confirmed genetically predicted lower *ACE* expression was associated with reduced SBP (β = −2.88 mmHg per SD decrease, 95 %CI −3.53 to −2.23 mmHg, p < 0.0001), demonstrating that the expression-based instrument captures biologically relevant ACE pathway variation. As a positive control, genetically predicted ACE inhibition was expectedly associated with lower risk of coronary artery disease (OR 0.90 per mmHg lower SBP, 95 %CI 0.85–0.96, p = 0.0004).

**Fig. 1:**
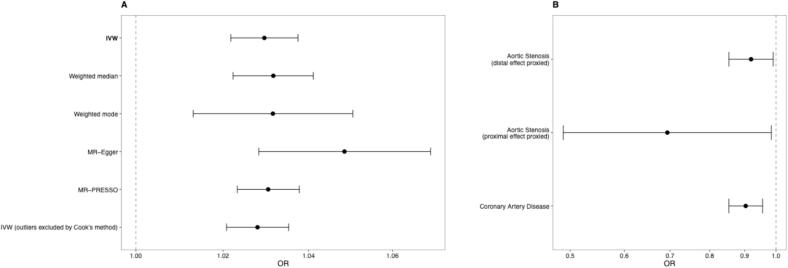
**A.** Conventional Mendelian randomization (MR) analyses for the genetically predicted effect of 1 mmHg increase in blood pressure on risk of aortic stenosis. Primary inverse variance-weighted (IVW) and sensitivity analyses are presented. **B.** Drug-target MR analyses for the effect of angiotensin converting enzyme (ACE) inhibition on risk of aortic stenosis proxied through its effects on systolic blood pressure (distal effect, Wald ratio analysis) and gene expression (proximal effect, summary-data based MR); and the genetically predicted distal effect of ACE inhibition on risk of coronary artery disease.

This drug-target MR study provides genetic evidence supporting a potential therapeutic role for ACE inhibitors in reducing the risk of AS. These results align with the biological understanding of AS pathogenesis, where elevated blood pressure may contribute to mechanical stress and valve calcification. The potential antifibrotic effects of ACE inhibition may further contribute to reduced risk of developing AS. These findings have important clinical implications, particularly given the current lack of medical therapies for AS and the widespread availability of ACE inhibitors. While our study cannot distinguish between AS initiation and progression, the results suggest clinical benefit is likely to be seen from early initiation and sustained administration of ACE inhibition. These results align with observational and MR studies demonstrating association between elevated blood pressure and AS risk, and observational data demonstrating improved survival with renin-angiotensin pathway blockade for patients with AS [1].

Several limitations warrant consideration. While sample overlap between the SBP and AS GWAS (particularly due to inclusion of UK Biobank data in both) could bias the distal drug-target MR result toward positive findings, our confidence is strengthened by concordant results from the proximal eQTL approach, which has minimal overlap with the AS GWAS. The consistent protective associations through both distal (OR 0.92) and proximal (OR 0.69) approaches suggest genuine biological effects rather than overlap artifacts. Additionally, the availability of a single SNP in the distal analysis precluded sensitivity analyses for horizontal pleiotropy that require multiple variants. The use of a biologically motivated *cis*-MR design is expected to reduce the risk of horizontal pleiotropy, although notably this cannot capture compound-specific off-target effects that occur with ACE inhibitor medications. Finally MR studies presume lifelong exposure from birth and therefore cannot inform optimal timing of drug therapy initiation [8].

This drug-target Mendelian randomization study provides genetic evidence supporting a potential role for ACE inhibition in reducing AS risk, with consistent results from both proximal gene expression and distal blood pressure approaches. Although genetic evidence cannot substitute for clinical trials, our results provide strong rationale for investigating ACE inhibitors in prospective randomized studies.

## Funding statement

J.C. reports grant funding from Heart Research Australia.

## CRediT authorship contribution statement

**Jonathan L. Ciofani:** Writing – review & editing, Writing – original draft, Visualization, Validation, Software, Resources, Project administration, Methodology, Investigation, Funding acquisition, Formal analysis, Data curation, Conceptualization. **Daniel Han:** Writing – review & editing, Visualization, Validation, Methodology, Formal analysis, Data curation, Conceptualization. **Benjamin Woolf:** Writing – review & editing, Validation, Software, Methodology. **Dipender Gill:** Writing – review & editing, Validation, Software, Methodology, Investigation, Formal analysis. **Usaid K. Allahwala:** Writing – review & editing, Supervision, Resources, Investigation. **Ravinay Bhindi:** Writing – review & editing, Supervision, Project administration, Investigation.

## Declaration of competing interest

The author declare the following financial interests/personal relationships which may be considered as potential competing interests: D.G. is CEO of Sequoia Genetics, a private company working with investors, pharma, biotech, and academia leveraging genetic data for drug discovery and development. D.G. has financial interests in several biotechnology companies.

## References

[1] Otto C.M., Nishimura R.A., Bonow R.O. (2021). 2020 ACC/AHA guideline for the management of patients with valvular heart disease: a report of the American college of cardiology/American heart association joint committee on clinical practice guidelines. Circulation.

[2] Ciofani J.L., Han D., Rao K. (2024). Lipid lowering therapies for aortic stenosis: a drug-target Mendelian randomisation study. Eur. Heart J. Cardiovasc. Pharmacother..

[3] Ciofani J.L., Han D., Allahwala U.K., Woolf B., Gill D., Bhindi R. (2023). Lipids, blood pressure, and diabetes mellitus on risk of cardiovascular diseases in East Asians: a mendelian randomization study. Am. J. Cardiol..

[4] Keaton J.M., Kamali Z., Xie T. (2024). Genome-wide analysis in over 1 million individuals of European ancestry yields improved polygenic risk scores for blood pressure traits. Nat. Genet..

[5] Theriault S., Li Z., Abner E. (2024). Integrative genomic analyses identify candidate causal genes for calcific aortic valve stenosis involving tissue-specific regulation. Nat. Commun..

[6] Nikpay M., Goel A., Won H.H. (2015). A comprehensive 1,000 Genomes-based genome-wide association meta-analysis of coronary artery disease. Nat. Genet..

[7] Vosa U., Claringbould A., Westra H.J. (2021). Large-scale cis- and trans-eQTL analyses identify thousands of genetic loci and polygenic scores that regulate blood gene expression. Nat. Genet..

[8] Ciofani J.L., Bhindi R. (2025). Timing of medical therapy for aortic stenosis: too little too late?. J. Am. Coll. Cardiol..

